# Predictive entrainment of natural speech through two fronto-motor top-down channels

**DOI:** 10.1080/23273798.2018.1506589

**Published:** 2018-09-26

**Authors:** Hyojin Park, Gregor Thut, Joachim Gross

**Affiliations:** a School of Psychology & Centre for Human Brain Health (CHBH), University of Birmingham, Birmingham, UK; b Institute for Biomagnetism and Biosignalanalysis, University of Muenster, Muenster, Germany; c Institute of Neuroscience and Psychology, University of Glasgow, Glasgow, UK

**Keywords:** Speech entrainment, top-down prediction, beta, delta

## Abstract

Natural communication between interlocutors is enabled by the ability to predict upcoming speech in a given context. Previously we showed that these predictions rely on a fronto-motor top-down control of low-frequency oscillations in auditory-temporal brain areas that track intelligible speech. However, a comprehensive spatio-temporal characterisation of this effect is still missing. Here, we applied transfer entropy to source-localised MEG data during continuous speech perception. First, at low frequencies (1–4 Hz, brain delta phase to speech delta phase), predictive effects start in left fronto-motor regions and progress to right temporal regions. Second, at higher frequencies (14–18 Hz, brain beta power to speech delta phase), predictive patterns show a transition from left inferior frontal gyrus via left precentral gyrus to left primary auditory areas. Our results suggest a progression of prediction processes from higher-order to early sensory areas in at least two different frequency channels.

## Introduction

Natural communication between interlocutors may seem effortless, however it relies on a series of complex computational tasks that have to be performed in the human brain in real-time and often in the presence of noise and other interferences. This high performance can only be achieved with the help of very effective prediction mechanisms (Levinson, 2016; Norris, McQueen, & Cutler, 2016). As auditory speech signals enter the sensory auditory system and are complemented by visual signals and cues, the human brain generates and constantly updates predictions about the timing and content of upcoming speech (Friston & Frith, 2015; Pickering & Garrod, 2007, 2013). In this context of natural conversation, we can model human brains as dynamic systems that are coupled through sensory information and operate according to active inference principles (Friston & Frith, 2015). In this framework, the brain relies on internal models to generate predictions about itself and others and updates the internal model to minimise prediction errors.

The temporal structure of this predictive coding mechanism can be mediated by cortical oscillations (Lakatos, Karmos, Mehta, Ulbert, & Schroeder, 2008) and previous studies have shown the computational role of cortical oscillations in speech processing as critical elements for parsing and segmentation of connected speech not only for auditory speech (Arnal & Giraud, 2012; Ding, Melloni, Zhang, Tian, & Poeppel, 2016; Giraud & Poeppel, 2012) but also for visual speech (Giordano et al., 2017; Park, Kayser, Thut, & Gross, 2016; Zion Golumbic, Cogan, Schroeder, & Poeppel, 2013). In addition, cortical oscillations track hierarchical components of speech rhythm and cortical oscillations themselves are hierarchically nested for speech tracking (Gross, Hoogenboom, et al., 2013). Importantly, these findings are evident only for intelligible speech processing where top-down modulation by prediction is possible. In our previous study, we found that low-frequency rhythms in the left frontal and motor cortices carry top-down signals to sensory areas, particularly to left auditory cortex, and this top-down signal was correlated with entrainment to speech (Park, Ince, Schyns, Thut, & Gross, 2015).

While previous studies have provided convincing evidence that low-frequency brain rhythms are involved in mediating top-down predictions, several important questions are still unresolved. First, what is the spatio-temporal structure of these prediction processes, or put differently, when and where in the brain is neural activity predictive of upcoming speech in an intelligibility-dependent manner? Second, what is the relationship between these low-frequency rhythms and higher frequency rhythms that have been implicated in prediction (Morillon & Baillet, 2017)? Third, how do predictive processes (preceding speech) interact with reactive processes (following speech) in temporally unfolding environment?

In order to address these questions, we used causal connectivity analysis – transfer entropy (TE) – to identify directed coupling between brain rhythms and speech rhythm for a range of positive delays (brain activity following speech) and negative delays (brain activity preceding speech) on the previously published dataset (Friston & Frith, 2015; Park et al., 2015). For the speech signal, we analysed low-frequency phase information which is a dominant spectral component in amplitude envelope of speech signal (Chandrasekaran, Trubanova, Stillittano, Caplier, & Ghazanfar, 2009). For the brain signal, we analysed both low-frequency phase information as well as high frequency beta power. We hypothesised that beta rhythms in the brain, particularly in higher order areas, are involved in the prediction of forthcoming speech as suggested by the role of beta oscillations in top-down predictive mechanism (Arnal & Giraud, 2012; Bastos et al., 2012; Fontolan, Morillon, Liegeois-Chauvel, & Giraud, 2014). A recent EEG study that used time-compressed speech reported beta oscillations reflecting an endogenous top-down channel which gradually builds up contextual information across time (Pefkou, Arnal, Fontolan, & Giraud, 2017). Particularly, beta oscillations in motor system were shown to be associated with precise temporal anticipation of forthcoming auditory inputs (Morillon & Baillet, 2017). We also hypothesised that this top-down “predictive speech coding” mechanism by beta oscillations (which should be represented at negative delays between brain activity and speech) recurrently interacts with low-frequency “speech entrainment” (represented at positive delays) where better prediction leads to stronger entrainment.

## Materials and methods

### Participants and experiment

Twenty-two volunteers participated in the study (11 females; age range 19–44 years, mean age ± SD: 27.2 ± 8.0 years). None of the participants had a history of psychological, neurological, or developmental disorders. They all had normal or corrected-to-normal vision and were right-handed. Written informed consent was obtained from all participants prior to the experiment and all participants received monetary compensation for their participation. The study was approved by the local ethics committee (FIMS00733; University of Glasgow, Faculty of Information and Mathematical Sciences) and conducted in accordance with the Declaration of Helsinki.

Participants were instructed to listen to a recording of a 7-min-long story (“Pie-man,” told by Jim O’Grady at “The Moth” storytelling event, New York). The stimulus was presented binaurally via a sound pressure transducer through two 5-meter-long plastic tubes terminating in plastic insert earpieces. Stimulus presentation was controlled via Psychtoolbox (Brainard, 1997) in MATLAB (MathWorks, Natick, MA). The experiment consisted of two conditions: standard (forward) presentation of story and backward played presentation of story. Experimental conditions were presented in randomised order across participants. Other analyses of these data have been published (Gross, Hoogenboom, et al., 2013; Park et al., 2015).

### Data acquisition, preprocessing and source localisation

For speech signal, we computed amplitude envelope (Chandrasekaran et al., 2009) using Chimera toolbox (Smith, Delgutte, & Oxenham, 2002). We first constructed nine frequency bands in the range 100–10,000 Hz to be equidistant on the cochlear map. The speech waveform was band-pass filtered in these bands using a fourth-order Butterworth filter (forward and reverse). Hilbert transform was applied for each band and amplitude envelopes were computed as absolute values. These amplitude envelopes were averaged across bands to obtain a wideband amplitude envelope that was used for all further analysis.

Data recordings were acquired with a 248-magnetometers whole-head MEG system (MAGNES 3600 WH, 4-D Neuroimaging) in a magnetically shielded room. Data were sampled at 1017 Hz and resampled at 250 Hz, denoised with information from the reference sensors, and detrended. The analysis was performed using the FieldTrip toolbox (Oostenveld, Fries, Maris, & Schoffelen, 2011) (http://fieldtrip.fcdonders.nl) and in-house MATLAB scripts according to the guidelines (Gross, Baillet, et al., 2013).

Structural T1-weighted magnetic resonance images (MRI) of each participant were obtained at 3T Siemens Trio Tim Scanner (Siemens, Erlangen, Germany) and co-registered to the MEG coordinate system using a semi-automatic procedure. Anatomical landmarks (nasion, left and right pre-auricular points) were manually identified in the individual’s MRI. Both coordinate systems were initially aligned based on these three points. Numerical optimisation by using the iterative closest point (ICP) algorithm (Besl & McKay, 1992) was applied.

Individual MRIs were segmented to grey matter, white matter, and cerebrospinal fluid to create individual head models. Leadfield computation was based on a single shell volume conductor model (Nolte, 2003) using a 10-mm grid defined on the standard template brain (Montreal Neurological Institute; MNI). The template grid was transformed into individual head space by linear spatial transformation. Cross-spectral density matrices were computed using Fast Fourier Transform on 1-s segments of data after applying Hanning window.

Frequency-specific spatial filters were computed for delta (1–4 Hz) and beta (14–18 Hz) bands at each voxel. We computed the covariance matrix over the full broad-band 7-minute data to compute LCMV filters for each voxel using 7% regularisation. These time series were then subjected to band-pass filtering (4th order Butterworth filter, forward and reverse). Dominant dipole orientation was estimated using SVD (singular value decomposition) at each voxel. Bandpass filtered data (1–4 Hz for delta band and 14–18 Hz for beta band) were projected through the filter to obtain band-limited time-series for each voxel. This computation was performed for each frequency band, and each experimental condition (forward and backward). Hilbert transformation was applied to the bandpass filtered data at each voxel to obtain instantaneous phase and power. We also used regions of interest (ROI) maps from the AAL (Automated Anatomical Labeling) atlas (Tzourio-Mazoyer et al., 2002) in order to delineate the temporal characteristics of directed causal relationship over the delays (see below) within the anatomically parcellated regions. We used ROIs labelled Heschl gyrus, inferior frontal gyrus – opercular part, and precentral gyrus.

### Directed causal connectivity analysis by transfer entropy (TE)

In this paper, we aimed to investigate two relationships of speech entrainment and predictive speech coding between speech and brain signal. In order to assess the relationships, we used transfer entropy (TE) that quantifies directed statistical dependencies between two signals, i.e. time-lagged predictability. TE is also known as Directed Information (Ince, Schultz, & Panzeri, 2014; Massey, 1990; Schreiber, 2000). Transfer entropy and Granger causality (Granger, 1969) conceptually similar and they are even identical for Gaussian data (Barnett, Barrett, & Seth, 2009) that we would expect to see very similar results from both methods. We used TE which is based on information theory in order to be consistent with our previous work (Park et al., 2015).

For speech entrainment, we computed TE from speech signal to signal at each brain voxel to quantify to what extent knowledge of speech signal reduces uncertainty in predicting the future of brain signal over and above what could be predicted from knowledge of the past of brain signal alone. For predictive speech coding, we computed TE from brain signal at each voxel to speech signal to quantify to what extent knowledge of brain signal reduces uncertainty in predicting the future of speech signal over and above what could be predicted from knowledge of the past of speech signal alone.

Specifically, we quantised the phase values from the two signals across all time points during stimulus presentation, and then used 4 bins in which each bin was equally occupied. For a specific delay d, we computed TE from speech (*X*) to brain (*Y*) for speech entrainment, and from brain (*X*) to speech (*Y*) for predictive speech coding as follows:

$$\begin{array}{rl} TE_{d}(X\to Y) & =CMI(X_{d};Y|Y_{d})\\ & =H(X_{d},Y_{d})+H(Y,Y_{d})-H(X_{d},Y,Y_{d})-H(Y_{d}) \end{array}$$

Where CMI is conditional mutual information, *H* represents entropy. The suffix *d* represents that signal is delayed with respect to the target signal *Y* by *d* milliseconds (i.e. considers that signal *d* milliseconds prior to *Y*). We computed entropy terms from the standard formula:

$$H(Y,Y_{d})=\sum_{a,b=1}^{4}\;p_{Y,Y_{d}}(a,b)\log_{2}p_{Y,Y_{d}}(a,b)$$

Where the joint distribution $p_{Y,Y_{d}}(a,b)$ is obtained from the multinomial maximum likelihood estimate obtained over time points:

$$p_{Y,Y_{d}}(a,b)=\sum_{t=d}^{Nt}\frac{\delta_{a}(Y(t))\delta_{b}(Y(t-d))}{Nt}$$

With $\delta_{a}(Y(t))$ a Kronecker delta function taking the value 1 if the binned phase value at *Y*(*t*) is quantile a and 0 otherwise.

In the TE computation bias correction was not applied. Bias of mutual information depends on the number of bins and time points that are used in the analysis (Panzeri, Senatore, Montemurro, & Petersen, 2007). In our analysis, we performed statistical contrast between conditions in which the same number of bins and time points was used for each calculation (Ince, Mazzoni, Bartels, Logothetis, & Panzeri, 2012). Bias correction reduces bias but increases the variance of the estimator, so comparisons between calculations with the same bias are better with uncorrected estimates.

The TE calculation was repeated for 25 different delays, from 20 to 500 ms with a 20-ms step. These computations were performed for each participant, and both conditions (forward, backward). For TE computation to study speech entrainment (TE from speech to brain), we analysed the same frequency (delta; 1–4 Hz) phase information for both speech and brain signals. For TE computation to study predictive speech coding (TE from brain to speech), we analysed 1) the same delta (1–4 Hz) phase information for both brain and speech signals as well as 2) beta (14–18 Hz) power for brain signal and delta (1–4 Hz) phase for speech signal. These computations resulted in TE values for each voxel, each delay, and each condition within each participant and then yielded to statistical comparison between conditions for each delay. For ROI analysis using AAL atlas map, TE values were first averaged across the voxels within the ROI, and then yielded to statistics.

Group statistics was performed using non-parametric randomisation in FieldTrip (Monte Carlo randomisation). Specifically, individual volumetric maps were smoothed with a 10-mm Gaussian kernel and subjected to dependent-samples *t*-test between conditions (forward versus backward). The null distribution was estimated using 500 randomisations and multiple comparison correction was performed using FDR (false discovery rate). Only significant results (*p* < 0.05, FDR-corrected) are reported.

## Results

To investigate the bidirectional nature of speech-brain coupling during listening to continuous speech we computed transfer entropy (TE) – an information-theoretic measure of directed causal connectivity – between speech and brain signals at positive and negative delays. This allows a disambiguation of two different effects that contribute to speech-brain coupling – namely those that follow speech (entrainment) and those that precede speech (prediction).

**Figure 1.**
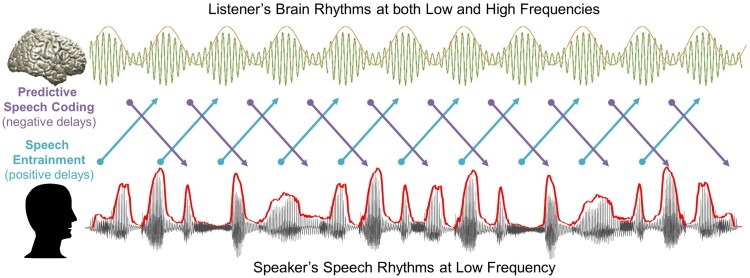
A schematic figure for temporal dynamics of information flow during natural speech perception. In the present study, we analysed transfer entropy (TE) to investigate two mechanisms of information flow during natural speech perception: (1) Predictive speech coding (top-down prediction mechanism investigated by negative delays between speech-brain; purple colour) and (2) Speech entrainment (stimulus-driven bottom-up processing investigated by positive delays between speech-brain; cyan colour). For speech signal, we used low-frequency delta phase information, and for brain signal, we used both low-frequency delta phase and high-frequency beta power information. Note that the signal waveform for the MEG signal shown in this figure and following figures is schematic illustration.

Since speech-brain coupling is strongest for the low frequency delta rhythm (1–4 Hz) we performed our analysis for this frequency band. In the following, we first present results for positive delays where delta-band brain activity follows speech. Second, we show how delta-band brain activity at different negative delays (i.e. preceding speech) in different brain areas predicts (in a statistical sense) the speech signal. Finally, since beta oscillations in motor cortex have been implicated in temporal predictions (e.g. (Arnal & Giraud, 2012; Morillon & Baillet, 2017) we investigate how these higher frequency oscillations in the brain are related to the low-frequency delta rhythm in speech (Figure 1).

## Entrained brain signals following speech (positive delays)

**Figure 2.**
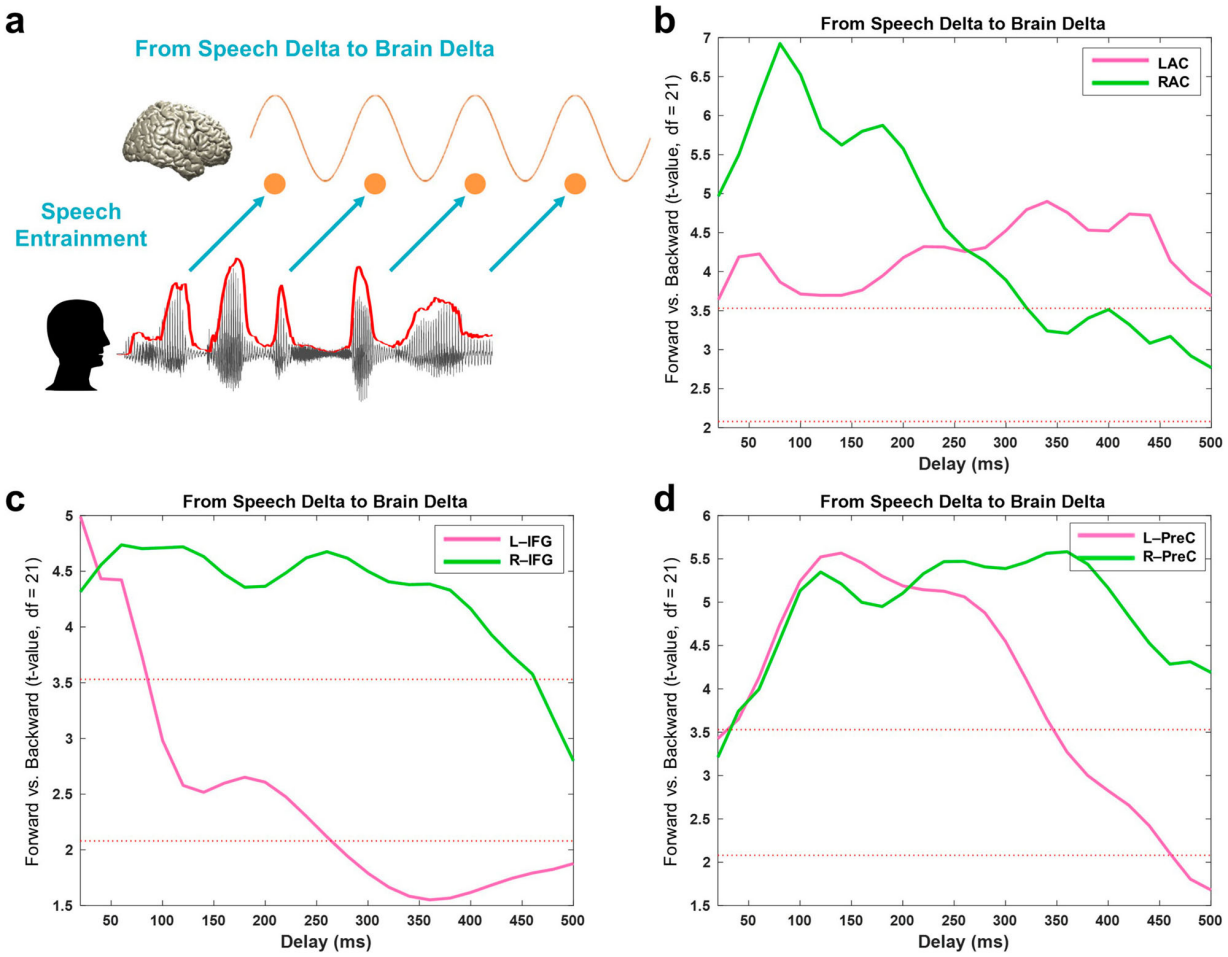
Entrained brain signals following speech. (a) A schematic figure for directed causal analysis: TE from speech delta phase to brain delta phase (positive delays between speech-brain). TE computation was performed for each condition (forward played and backward played) at each voxel from 20 to 500 ms with a 20-ms step. Orange line represents delta rhythm in the brain and each circle represents a certain point in time. TE values are averaged within each ROI from the AAL atlas and compared statistically between conditions. *T*-values are shown in each ROI bilaterally (pink: left hemisphere, green: right hemisphere): (b) primary auditory cortex (Heschl gyrus) (c) Inferior frontal gyrus – opercular part (BA44) (d) precentral gyrus. Statistical significance was shown with two red lines depicting *t*-values by paired *t*-test (upper red line: *t*_21_ = 3.53, *p* < 0.05, corrected; bottom red line: *t*_21_ = 2.08, *p* < 0.05, uncorrected).

We first examine how low-frequency speech signal entrains the same frequency brain rhythms with positive delays (brain signals following speech signals, Figure 2a). We computed TE from speech envelope to brain signals from different ROIs with delays ranging from 20 to 500 ms in steps of 20 ms. Based on our recent study (Park et al., 2015), we focused on three ROIs from the AAL atlas; primary auditory cortex, inferior frontal gyrus (IFG) and precentral gyrus. We computed TE between forward and backward played speech conditions (Figure 2b–d). Whole brain results of the same analysis are shown in Supplementary Figure 1.

Figure 2b shows the statistical difference between forward and backward played speech across different delays for left (pink) and right (green) auditory cortex. Highest *t*-values are observed at delays of about 80 ms in right auditory cortex and are stronger than corresponding effects in left auditory cortex. The effect of intelligibility remains significant for delays up to about 300 ms for right auditory cortex and for delays up to 500 ms for left auditory cortex. In IFG, the effect of intelligibility is also most pronounced in the right hemisphere (up to about 450 ms) compared to only early transient effect for the left hemisphere (Figure 2c). Precentral areas in left and right hemisphere show similar sensitivity to intelligibility but are longer lasting in the right hemisphere (Figure 2d) (Figure 2b-d: paired *t*-test; upper red line: *t*_21_ = 3.53, *p* < 0.05, corrected; bottom red line: *t*_21_ = 2.08, *p* < 0.05, uncorrected).

## Entrained brain signals predicting upcoming speech (negative delays)

**Figure 3.**
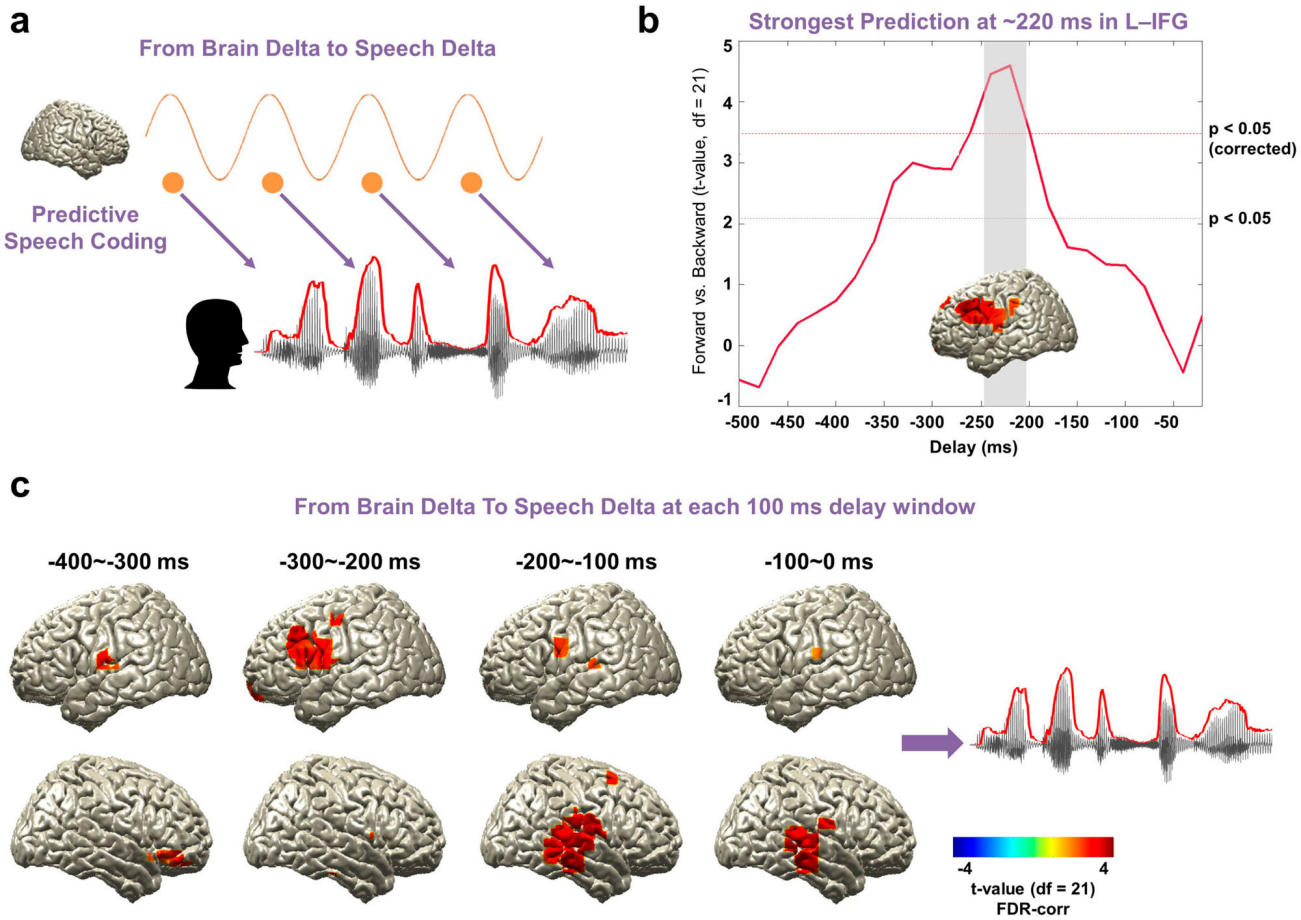
Entrained brain signals predicting upcoming speech. (a) A schematic figure for directed causal analysis: TE from brain delta phase to speech delta phase (negative delays between speech-brain). TE computation was performed for each condition (forward played and backward played) at each voxel from 20 to 500 ms in 20-ms steps and compared statistically between conditions. Orange line represents delta rhythm in the brain and each circle represents a certain point in time. (b) The strongest prediction was found at ∼220 ms in left IFG (upper red line: *t*_21_ = 3.53, *p* < 0.05, corrected; bottom red line: *t*_21_ = 2.08, *p* < 0.05, uncorrected). (c) Statistical contrast maps of averaged across 100 ms windows show a sequence of events that start around −300 ms in fronto-motor areas and then move to right auditory-temporal areas at around −200 ms prior to speech (*p* < 0.05, FDR-corrected).

Next, we aimed to characterise the spatio-temporal pattern of predictive processes in the brain in the same low-frequency band (1–4 Hz, Figure 3a). Specifically, we computed TE between speech and brain signals over a range of negative delays (−500 ms to −20 ms in steps of 20 ms) to assess where and when brain signals predict significantly stronger upcoming forward compared to backward speech. We performed the computation in the whole brain and show maps of statistical difference between forward and backward speech condition. Overall, we found the strongest directed effect in left fronto-motor regions ∼−220 ms prior to the forthcoming speech when speech is played forward compared to backward (Figure 3b: paired *t*-test; upper red line: *t*_21_ = 3.53, *p* < 0.05, corrected; bottom red line: *t*_21_ = 2.08, *p* < 0.05, uncorrected).

To further characterise the spatio-temporal evolution of brain signals that predict upcoming speech, we averaged TE-maps in 100 ms windows and computed again the statistical contrast of forward compared to backward played speech (Figure 3c). This revealed a sequence of events that starts around −300 ms in fronto-motor areas (see also Figure 3b) and then moves to right auditory-temporal areas at around −200 ms prior to speech (Figure 3c: *p* < 0.05, FDR-corrected). These results indicate that brain activity in fronto-motor areas preceding speech by 300 ms contain information that predicts the forthcoming speech significantly better for forward compared to backward speech. The same holds true for right auditory-temporal areas at shorter delays of 200 ms preceding speech. This suggests that predictive speech mechanisms occur first in left inferior fronto-motor areas and later in right auditory-temporal areas.

## Beta rhythms and the prediction of upcoming speech (negative delays)

**Figure 4.**
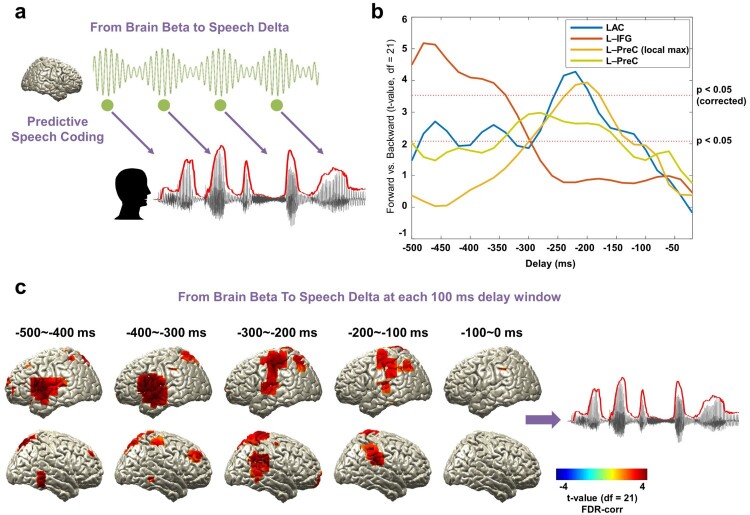
Beta rhythms and the prediction of upcoming speech. (a) A schematic figure for directed causal analysis: TE from brain beta power to speech delta phase (negative delays between speech-brain). TE computation was performed for each condition (forward played and backward played) at each voxel from 20 to 500 ms in 20 ms steps and compared statistically between conditions. Green line represents beta rhythm in the brain and each circle represents a certain point in time. (b) TE values are averaged within each ROI from the AAL atlas and compared statistically between conditions. *T*-values are shown in each ROI: left primary auditory cortex (Heschl gyrus) (blue line), left IFG (orange line), and left precentral gyrus (both at local maximum coordinate (yellow line) and whole precentral gyrus ROI (light green line)). Statistical significance was shown with two red lines depicting *t*-values by paired *t*-test (upper red line: *t*_21_ = 3.53, *p* < 0.05, corrected; bottom red line: *t*_21_ = 2.08, *p* < 0.05, uncorrected). Left-lateralised predictions by beta power were observed (see Supplementary Figure 2). (c) Statistical contrast maps of averaged across 100 ms windows show that intelligibility-dependent prediction first engages left inferior frontal gyrus about 300–500 ms prior to the speech followed by left precentral gyrus and left primary auditory area 200–300 ms prior to the speech (*p* < 0.05, FDR-corrected).

As we hypothesised based on the literature that beta rhythms are involved in predictive process during speech processing, we examined the causal relationship between beta rhythm in the brain and low-frequency rhythm in the speech signal. We computed TE from beta (14–18 Hz) power at each voxel to the low-frequency delta (1–4 Hz) phase of the speech signal for a range of delays (−500 ms to −20 ms in steps of 20 ms, Figure 4a). The same computation was performed for the forward and backward conditions and then statistically compared. We first focus on the temporal dynamics of the three ROIs (primary auditory cortex (Heschl gyrus), inferior frontal gyrus, precentral gyrus).

Figure 4b shows over a range of delays for each ROI to what extent the prediction of forthcoming speech depends on speech intelligibility.

Beta power in left inferior frontal gyrus shows strong intelligibility-dependent prediction about 340–500 ms prior to speech (Figure 4b orange line). Left primary auditory cortex and left precentral gyrus show a similar significant effect but at a shorter delay peaking just before −200 ms (Figure 4b, blue and yellow lines). For the precentral gyrus, the ROI map from the AAL atlas is rather big, so we extracted the time series for the voxel with the local maximum in this area (yellow line, but also see the similar pattern for whole precentral gyrus ROI in the AAL atlas; light green line). Interestingly, this intelligibility-dependent prediction in these ROIs was left-lateralized (see Figure 4c and Supplementary Figure 2) (Figure 4b: paired *t*-test; upper red line: *t*_21_ = 3.53, *p* < 0.05, corrected; bottom red line: *t*_21_ = 2.08, *p* < 0.05, uncorrected). Whole brain results averaged across 100 ms-long windows corroborated this pattern that first engages left inferior frontal gyrus about 300–500 ms prior to the speech followed by left precentral gyrus and left primary auditory area 200–300 ms prior to the speech (Figure 4c: *p* < 0.05, FDR-corrected).

We have analysed predictive mechanism on speech theta rhythm (4–7 Hz) which represents syllabic rate (Alexandrou, Saarinen, Kujala, & Salmelin, 2016; Giraud & Poeppel, 2012) however, we did not find any significant results. This might be due to the fact that both word rate (2.3 Hz) and syllable rate (3.0 Hz) in our speech stimuli fall into delta rhythm. This was revealed by a transcription analysis (959 words and 1237 syllables during 420 s) and word production rate matches previous findings (Alexandrou et al., 2016; Levelt, Roelofs, & Meyer, 1999; Ruspantini et al., 2012).

## Relationship between temporal dynamics of speech entrainment and predictive speech coding

We next assessed how intelligibility-dependent prediction and the temporal dynamics of speech entrainment are related. We hypothesised that predictive control mechanisms interact with the rhythms in the brain driven by speech. We performed correlation analysis between TE values of intelligible speech at negative delays (preceding speech) and positive delays (following speech) using robust Spearman rank correlations across participants (Pernet, Wilcox, & Rousselet, 2013).

Motivated by the role of top-down beta activity particularly in the perception of sustained temporal aspect of speech (Pefkou et al., 2017), we correlated predictive coding by beta activity (Figure 4) and speech entrainment (Figure 2) at various delays. We studied this mechanism first within early sensory area (auditory cortex) as well as higher order areas where we found strong top-down prediction.

**Figure 5.**
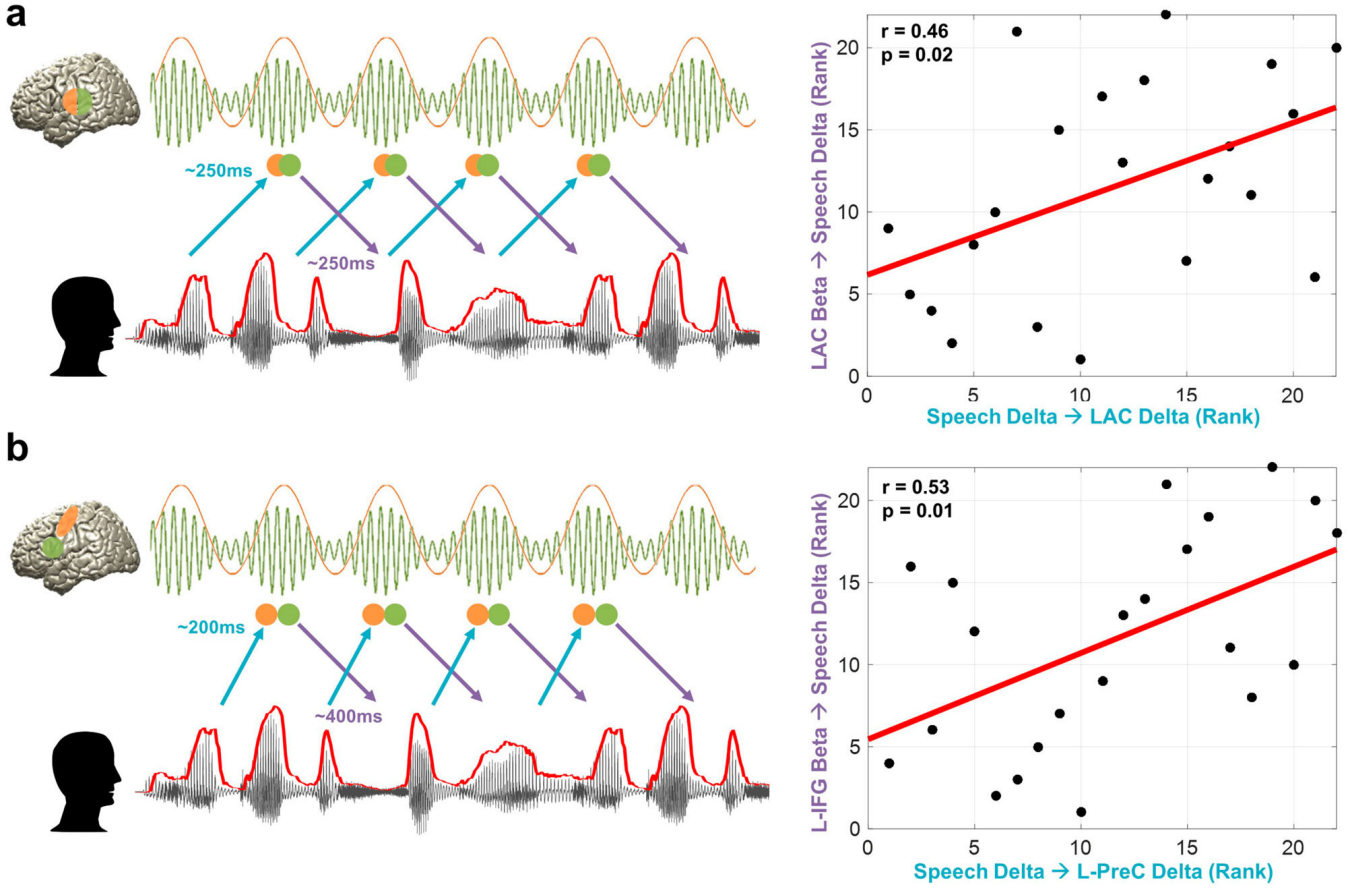
Relationship between temporal dynamics of speech entrainment and predictive speech coding. To assess how the temporal dynamics of speech entrainment (positive delays) and intelligibility-dependent top-down prediction (negative delays) are interacting, we performed correlation analysis using robust Spearman rank correlations across participants between the two mechanisms (but for top-down prediction, we used TE from brain beta to speech delta; Figure 4). Orange and green colours represent delta and beta rhythms in the brain and each circle represents a certain point in time. We tested the relationship within in early sensory area. i.e. primary auditory cortex as well as higher order areas. (a) Delta phase in the left auditory cortex driven by speech (corresponding to the plot for the left auditory cortex (pink line) at ∼250 ms in the Figure 2b) is associated with predictive speech coding mechanism of beta power in the left auditory cortex ∼250 ms prior to the forthcoming speech with low frequency delta phase information (corresponding to the plot for the left auditory cortex (blue line) at ∼250 ms in the Figure 4b) (*r* = 0.46, *p* = 0.02). (b) Delta phase in the left precentral gyrus modulated by the same frequency phase in the speech at ∼200 ms (corresponding to the plot for the left precentral gyrus (pink line) at ∼200 ms in the Figure 2d) is associated with predictive speech coding of beta power in the left inferior frontal gyrus ∼400 ms prior to the forthcoming speech with low frequency delta phase information (corresponding to the plot for left inferior frontal gyrus (orange line) at ∼400 ms in the Figure 4b) (*r* = 0.53, *p* = 0.01).

Figure 5a shows this relationship in the left primary auditory cortex where predictive speech coding mechanism of beta power in the left auditory cortex ∼250 ms prior to the forthcoming speech with low frequency delta phase information (corresponding to the plot for the left auditory cortex (blue line) at ∼250 ms in the Figure 4b) is closely associated with low-frequency delta rhythm in the left auditory cortex driven by speech (corresponding to the plot for the left auditory cortex (pink line) at ∼250 ms in the Figure 2b) (*r* = 0.46, *p* = 0.02). This suggests that individuals with stronger predictive coding by beta power in the left auditory cortex ∼250 ms prior to the upcoming low frequency delta phase information in the speech signal are capable of stronger speech entrainment in the left auditory cortex by speech rhythm with low frequency delta phase at ∼250 ms.

Another interesting aspect of this relationship emerged between the left inferior frontal cortex and left precentral gyrus. Figure 5b shows that beta power in the left inferior frontal gyrus ∼400 ms prior to the forthcoming speech (delta phase) is associated with delta phase in the left precentral gyrus following the same frequency phase in the speech at ∼200 ms (corresponding to the plot for the left precentral gyrus (pink line) at ∼200 ms in the Figure 2d) (*r* = 0.53, *p* = 0.01). This is a relationship between predictive coding (negative delay; corresponding to the plot for left inferior frontal gyrus (orange line) at ∼400 ms in the Figure 4b) and reactive processing (positive delay; corresponding to the plot for the left precentral gyrus (pink line) at ∼200 ms in the Figure 2d). This suggests that individuals with stronger modulation of predictive coding by beta power in the left inferior frontal gyrus ∼400 ms prior to the low frequency delta phase information in the speech signal are capable of stronger speech entrainment in the left precentral gyrus by speech rhythm with low frequency delta phase at ∼200 ms.

## Discussion

Here, we aimed to study the spatio-temporal characteristics of predictions during continuous speech recognition. We analysed transfer entropy (TE) at various (positive and negative) delays between the speech envelope and brain activity. At low frequencies (1–4 Hz) our results reveal a progression of predictive effects from left fronto-motor regions to right temporal regions. A different pattern emerged when we investigated TE between beta power in the brain and the phase of 1–4 Hz components in the speech envelope. We first see an engagement of left inferior frontal gyrus about 300–500 ms prior to speech followed by left precentral gyrus and left primary auditory areas. Our results suggest a progression of prediction processes from higher-order to early sensory areas.

First, it is important to carefully consider what aspects of predictions are captured using our approach. Transfer entropy (TE) is an information theoretic measure that quantifies directed statistical dependencies between time series. Specifically, TE from signal *X* to signal *Y* quantifies to what extent knowledge of *X* reduces uncertainty in predicting the future of *Y* over and above what could be predicted from knowledge of the past of *Y* alone. TE is conceptually similar to Granger causality as it infers causal relationships from time-lagged predictability. Here, when analysing prediction effects, we quantify to what extent the past of brain activity in a certain brain area improves prediction of the future of the speech envelope (over and above what could be predicted from the past of the speech envelope alone). Our main conclusions are then based on the statistical contrast between forward played speech and backward played speech. This is important for two reasons. First, the power spectrum of the speech envelope is the same for both conditions. Therefore, this statistical contrast controls (to some extent) for the low-level rhythmicity in speech and amplifies sensitivity of the analysis to intelligibility. However, we acknowledge that backward played speech is not a perfect control condition due to differences in the finer temporal structure and attention to the stimulus. However, we acknowledge that backward played speech is not a perfect control condition due to differences in the finer temporal structure and attention to the stimulus. We assume that attention or predictions during presentation of backward played speech will not change systematically over time, therefore temporal profile of our TE results (TE changes over delays) is unlikely to be affected by attention. However we did not explicitly monitor the difference in attentional level between the conditions so we cannot rule out that condition-specific differences in attention affected the results. Second, similar to Granger causality, the computation of TE on bandpass-filtered data is not without problems (Florin, Gross, Pfeifer, Fink, & Timmermann, 2010; Weber, Florin, von Papen, & Timmermann, 2017). Statistically contrasting two conditions will counteract these problems. In addition, we would like to note that our results are very similar when using delayed mutual information (data not shown), a measure that is less sensitive to the effects of filtering. In summary, our approach is expected to be mostly sensitive to intelligibility-related components in speech.

Still, from our study it is difficult to exactly specify the structure in speech that is the target of the prediction processes presented here. This is in contrast to the many studies that demonstrate that a semantic violation at a certain point in a sentence gives rise to the well-known N400 evoked response (e.g. Kutas and Federmeier (2011)). Our main results show that both low (delta: 1–4 Hz) and high frequency (beta: 14–18 Hz) brain oscillations predict speech envelope in the delta band (1–4 Hz). Since we do not have controlled manipulations of linguistic content or temporal profile in our experiment, we could interpret the results based on what we have used in the analysis. By definition, higher TE values refer to a situation where prediction of the future of one time series *X* can be improved by knowing the past of another time series *Y* over and above predictions based on the past of time series *X* alone. The method is therefore sensitive to any information that is represented in the MEG and speech time series. Consequently, our TE effects could be driven by acoustic features, linguistic content or both. In combination with the statistical comparison (forward vs. backward speech conditions), we interpret that signal input that is related to lexical and phrasal units (delta band) in forward speech can be predicted. However, this is currently only speculation. This question should be elucidated with further experimental manipulations on different levels such as semantics, grammar, syntax, or linguistic contents. While being less controlled, our approach benefits from ecological validity and more directly taps into prediction processes that operate during natural speech processing.

Brain oscillations in the delta band (1–4 Hz) follow the same frequency in the speech envelope robustly across all delays between 20 and 500 ms. The pattern is stronger in the right hemisphere (higher *t*-values in the Supplementary Figure 1). In our previous study we showed more right-lateralised speech-brain coupling at a fixed delay using mutual information (Figure 2a in Gross, Hoogenboom, et al. (2013)). This mechanism seems to extend to the directed TE measure used here up to ∼400 ms delays. This supports the asymmetric sampling in time (AST) model (Poeppel, 2003) that posits a right hemisphere preference for longer temporal integration window (∼150–250 ms). The sustained pattern suggests brain responses modulated by speech envelope are critical to continuous intelligible speech perception.

Directed coupling between speech envelope and brain oscillations across negative delays suggests a predictive coding mechanism where sensory processing is modulated in top-down manner. We investigated this mechanism from low-frequency delta (1–4 Hz) phase in the brain to low-frequency delta phase in the speech envelope and from beta power (14–18 Hz) in the brain to low-frequency delta (1–4 Hz) phase in the speech envelope. Our study reveals robust prediction processes at low frequencies (1–4 Hz). This is the frequency that represents intonation and prosody (Ghitza, 2011; Giraud & Poeppel, 2012) but also overlaps at the upper end with the mean syllable rate (Ding et al., 2017). It shows an interesting temporal progression from left inferior fronto-motor areas to right auditory-temporal areas. This progression of prediction from higher order areas in the left hemisphere (200–300 ms prior to speech) to the early sensory areas in the right hemisphere suggests that the brain first generates prediction of upcoming sensory input by top-down contextual knowledge that is later used for optimised stimulus encoding in early sensory areas. Indeed, top-down modulations of delta phase (such as temporal expectation or selection of an attended stimulus stream) has been shown to increase sensitivity to external inputs in the auditory (Lakatos et al., 2008) and visual domain (Cravo, Rohenkohl, Wyart, & Nobre, 2013). Similarly, we find that beta power in the left frontal cortex and sensorimotor areas reflects prediction of upcoming speech relatively early (200–500 ms prior to speech) and is left-lateralised (Supplementary Figure 2). Active inference by motor systems regarding predictive coding has been studied recently and beta oscillation has been suggested to be working together with low-frequency activity in top-down modulation of ongoing activity during predictive coding (Arnal & Giraud, 2012). Recently an elegant study employing an auditory attention task has shown that interdependent delta and beta activity from left sensorimotor cortex encodes temporal prediction and this is directed towards auditory areas (Morillon & Baillet, 2017). This is consistent with our finding that both delta phase and beta power in the left frontal and sensorimotor engages in the prediction of forthcoming speech from relatively early stage. The temporal progression from inferior frontal to motor areas seems to suggest a hierarchical organisation of prediction processes that warrant further investigation. Based on the previous findings regarding potential top-down prediction effects from different frequency bands, including alpha frequency band (Rohenkohl & Nobre, 2011; Wostmann, Herrmann, Wilsch, & Obleser, 2015), we have computed TE for other frequency bands (alpha: 8–13 Hz, high beta: 19–30 Hz, and gamma: 35–45 Hz), however, we did not find any significant effects.

During continuous speech perception, brain oscillations entrained by speech (positive delays) and predicting speech (negative delays) are expected to interact in time. In other words, there is a continuous recurrent interaction between stimulus-driven bottom-up processing and top-down prediction processing during continuous speech perception. This enables the brain to estimate input statistics as well as calibrate top-down and bottom-up processing of temporally unfolding features (Hasson, 2017) such as speech in the present study.

We focused our analysis on the interaction between top-down predictive coding, i.e. TE from beta power in the brain to delta phase in the speech (Figure 4, negative delays) in the auditory cortex and higher order areas, i.e. fronto-motor areas and speech entrainment, i.e. TE from delta phase in the speech to delta phase in the brain (Figure 2, positive delays). In the left auditory cortex (Figure 5a), subjects with better top-down prediction by beta power at ∼250 ms (∼ peak of LAC in Figure 4b; Supplementary Figure 2b) show better entrainment by speech delta phase at ∼250 ms (∼crossing point between the LAC and RAC in Figure 2b). This result indicates that although low-frequency brain oscillations following speech envelope seems stronger in the right hemisphere than the left hemisphere (higher *t*-value for RAC at early delays in Figure 2b; Supplementary Figure 1), the interaction between both (top-down prediction and bottom-up speech entrainment) is modulated by the left auditory cortex (Park et al., 2015). In the higher order areas (Figure 5b), speech-driven bottom-up information flow to the left motor cortex in the delta phase at delays ∼200 ms is strongly associated with top-down predictive information flow from beta power in the left IFG to speech delta phase at ∼400 ms prior to the speech. This indicates that subjects with stronger top-down predictive speech coding ∼400 ms prior to the upcoming speech by beta power in the left IFG (orange line in Figure 4b; Supplementary Figure 2c) are also better bottom-up entrained by speech delta phase at ∼200 ms in the left motor cortex (pink line in Figure 2d).

It should be noted that the stimulus we used is a prepared and organised text (read-aloud text as a monologue speech) that was spoken at a storytelling event which enables more intelligible speech comprehension when compared to spontaneously produced speech which is characterised by disfluencies (e.g. interruptions, repetitions, false starts) (Hirose & Kawanami, 2002; Tree, 1995, 2001). Since it is unclear how our findings generalise across speech settings, studying possible differences in predictive mechanisms between different speech settings would be an interesting topic for future studies.

It also should be noted that our results from statistical comparison between standard speech and backward played speech could result from differences in relation to temporal structure of acoustic amplitude envelope other than speech intelligibility. Normal speech has fast onsets and long decays whereas reversed speech has the opposite pattern (slow onsets and rapid decays) (Narain et al., 2003), so it should be interpreted with caution. In order to reduce this concern, other types of control condition, e.g. foreign languages, would be required. In addition, the transcription analysis revealed that word and syllable rate overlap with the delta band. Therefore, our delta band results might reflect phase resetting driven due to word and syllable onsets.

From a broader perspective, it is worth asking whether processes involved in this predictive mechanism are specialised for speech or underlie general auditory processes including music perception.

In summary, our results indicate that predictive processes during continuous speech processing involve fronto-motor areas, operate in at least two frequency channels (delta and beta), follow an organised temporal progression from higher-order areas to early sensory areas and recurrently interact with reactive processes. Further research is needed to decode the exact nature of these predictions, identify the contributions of individual areas and elucidate the mutual dependencies between processes that precede and follow speech. In addition, in order to gain a better understanding of interaction between top-down predictive and bottom-up processing, studies using more complex type of stimuli that manipulate degrees of uncertainty (Hasson, 2017) would be helpful.

## Supplementary Material

PLCP_A_1506589_Supplemental Material

## References

[CIT0001] Alexandrou, A. M., Saarinen, T., Kujala, J., & Salmelin, R. (2016). A multimodal spectral approach to characterize rhythm in natural speech. *The Journal of the Acoustical Society of America*, *139*(1), 215–226. doi: 10.1121/1.4939496 26827019

[CIT0002] Arnal, L. H., & Giraud, A. L. (2012). Cortical oscillations and sensory predictions. *Trends in Cognitive Sciences*, *16*(7), 390–398. doi: 10.1016/j.tics.2012.05.003 22682813

[CIT0003] Barnett, L., Barrett, A. B., & Seth, A. K. (2009). Granger causality and transfer entropy are equivalent for Gaussian variables. *Physical Review Letters*, *103*(23), 238701. doi: 10.1103/PhysRevLett.103.238701 20366183

[CIT0004] Bastos, A. M., Usrey, W. M., Adams, R. A., Mangun, G. R., Fries, P., & Friston, K. J. (2012). Canonical microcircuits for predictive coding. *Neuron*, *76*(4), 695–711. doi: 10.1016/j.neuron.2012.10.038 23177956 PMC3777738

[CIT0005] Besl, P. J., & McKay, N. D. (1992). A method for registration of 3-D shapes. *IEEE Transactions on Pattern Analysis*, *14*, 239–256. doi: 10.1109/34.121791

[CIT0006] Brainard, D. H. (1997). The psychophysics toolbox. *Spatial Vision*, *10*(4), 433–436. doi: 10.1163/156856897X00357 9176952

[CIT0007] Chandrasekaran, C., Trubanova, A., Stillittano, S., Caplier, A., & Ghazanfar, A. A. (2009). The natural statistics of audiovisual speech. *PLoS Computational Biology*, *5*(7), e1000436. doi: 10.1371/journal.pcbi.1000436 19609344 PMC2700967

[CIT0008] Cravo, A. M., Rohenkohl, G., Wyart, V., & Nobre, A. C. (2013). Temporal expectation enhances contrast sensitivity by phase entrainment of low-frequency oscillations in visual cortex. *Journal of Neuroscience*, *33*(9), 4002–4010. doi: 10.1523/JNEUROSCI.4675-12.2013 23447609 PMC3638366

[CIT0009] Ding, N., Melloni, L., Zhang, H., Tian, X., & Poeppel, D. (2016). Cortical tracking of hierarchical linguistic structures in connected speech. *Nature Neuroscience*, *19*(1), 158–164. doi: 10.1038/nn.4186 26642090 PMC4809195

[CIT0010] Ding, N., Patel, A. D., Chen, L., Butler, H., Luo, C., & Poeppel, D. (2017). Temporal modulations in speech and music. *Neuroscience &amp; Biobehavioral Reviews*, *81*(Pt B), 181–187. doi: 10.1016/j.neubiorev.2017.02.011 28212857

[CIT0011] Florin, E., Gross, J., Pfeifer, J., Fink, G. R., & Timmermann, L. (2010). The effect of filtering on granger causality based multivariate causality measures. *Neuroimage*, *50*(2), 577–588. doi: 10.1016/j.neuroimage.2009.12.050 20026279

[CIT0012] Fontolan, L., Morillon, B., Liegeois-Chauvel, C., & Giraud, A. L. (2014). The contribution of frequency-specific activity to hierarchical information processing in the human auditory cortex. *Nature Communications*, *5*(4694), doi: 10.1038/ncomms5694 PMC4164774 25178489

[CIT0013] Friston, K. J., & Frith, C. D. (2015). Active inference, communication and hermeneutics. *Cortex*, *68*, 129–143. doi: 10.1016/j.cortex.2015.03.025 25957007 PMC4502445

[CIT0014] Ghitza, O. (2011). Linking speech perception and neurophysiology: Speech decoding guided by cascaded oscillators locked to the input rhythm. *Frontiers in Psychology*, *2*, 130. doi: 10.3389/fpsyg.2011.00130 21743809 PMC3127251

[CIT0015] Giordano, B. L., Ince, R. A. A., Gross, J., Schyns, P. G., Panzeri, S., & Kayser, C. (2017). Contributions of local speech encoding and functional connectivity to audio-visual speech perception. *Elife*, *6*, doi: 10.7554/eLife.24763 PMC5462535 28590903

[CIT0016] Giraud, A. L., & Poeppel, D. (2012). Cortical oscillations and speech processing: Emerging computational principles and operations. *Nature Neuroscience*, *15*(4), 511–517. doi: 10.1038/nn.3063 22426255 PMC4461038

[CIT0017] Granger, C. W. J. (1969). Investigating causal relations by econometric models and cross-spectral methods. *Econometrica*, *37*(3), 424–438. doi: 10.2307/1912791

[CIT0018] Gross, J., Baillet, S., Barnes, G. R., Henson, R. N., Hillebrand, A., Jensen, O., … Schoffelen, J. M. (2013). Good practice for conducting and reporting MEG research. *Neuroimage*, *65*, 349–363. doi: 10.1016/j.neuroimage.2012.10.001 23046981 PMC3925794

[CIT0019] Gross, J., Hoogenboom, N., Thut, G., Schyns, P., Panzeri, S., Belin, P., & Garrod, S. (2013). Speech rhythms and multiplexed oscillatory sensory coding in the human brain. *PLoS Biology*, *11*(12), e1001752. doi: 10.1371/journal.pbio.1001752 24391472 PMC3876971

[CIT0020] Hasson, U. (2017). The neurobiology of uncertainty: Implications for statistical learning. *Philosophical Transactions of the Royal Society B: Biological Sciences*, *372*(1711), doi: 10.1098/rstb.2016.0048 PMC5124074 27872367

[CIT0021] Hirose, K., & Kawanami, H. (2002). Temporal rate change of dialogue speech in prosodic units as compared to read speech. *Speech Communication*, *36*(1-2), 97–111. doi: 10.1016/S0167-6393(01)00028-0

[CIT0022] Ince, R. A., Mazzoni, A., Bartels, A., Logothetis, N. K., & Panzeri, S. (2012). A novel test to determine the significance of neural selectivity to single and multiple potentially correlated stimulus features. *Journal of Neuroscience Methods*, *210*(1), 49–65. doi: 10.1016/j.jneumeth.2011.11.013 22142889

[CIT0023] Ince, R. A., Schultz, S. R., & Panzeri, S. (2014). Estimating information-theoretic quantities. In D. Jaeger & R. Jung (Eds.), *Encyclopedia of computational neuroscience*.

[CIT0024] Kutas, M., & Federmeier, K. D. (2011). Thirty years and counting: Finding meaning in the N400 component of the event-related brain potential (ERP). *Annual Review of Psychology*, *62*, 621–647. doi: 10.1146/annurev.psych.093008.131123 PMC4052444 20809790

[CIT0025] Lakatos, P., Karmos, G., Mehta, A. D., Ulbert, I., & Schroeder, C. E. (2008). Entrainment of neuronal oscillations as a mechanism of attentional selection. *Science*, *320*(5872), 110–113. doi: 10.1126/science.1154735 18388295

[CIT0026] Levelt, W. J., Roelofs, A., & Meyer, A. S. (1999). A theory of lexical access in speech production. *Behavioral and Brain Sciences*, *22*(1), 1–38. discussion 38–75. 10.1017/s0140525x99001776 11301520

[CIT0027] Levinson, S. C. (2016). Turn-taking in human communication – origins and implications for language processing. *Trends in Cognitive Sciences*, *20*(1), 6–14. doi: 10.1016/j.tics.2015.10.010 26651245

[CIT0028] Massey, J. (1990). *Causality, feedback and directed information*. Proceedings International Symposium Information Theory Application (ISITA 1990), pp. 303–305.

[CIT0029] Morillon, B., & Baillet, S. (2017). Motor origin of temporal predictions in auditory attention. *Proceedings of the National Academy of Sciences*, *114*(42), E8913–E8921. doi: 10.1073/pnas.1705373114 PMC5651745 28973923

[CIT0030] Narain, C., Scott, S. K., Wise, R. J., Rosen, S., Leff, A., Iversen, S. D., & Matthews, P. M. (2003). Defining a left-lateralized response specific to intelligible speech using fMRI. *Cerebral Cortex*, *13*(12), 1362–1368. doi: 10.1093/cercor/bhg083 14615301

[CIT0031] Nolte, G. (2003). The magnetic lead field theorem in the quasi-static approximation and its use for magnetoencephalography forward calculation in realistic volume conductors. *Physics in Medicine and Biology*, *48*(22), 3637–3652. doi: 10.1088/0031-9155/48/22/002 14680264

[CIT0032] Norris, D., McQueen, J. M., & Cutler, A. (2016). Prediction, Bayesian inference and feedback in speech recognition. *Language, Cognition and Neuroscience*, *31*(1), 4–18. doi: 10.1080/23273798.2015.1081703 PMC4685608 26740960

[CIT0033] Oostenveld, R., Fries, P., Maris, E., & Schoffelen, J. M. (2011). Fieldtrip: Open source software for advanced analysis of MEG, EEG, and invasive electrophysiological data. *Computational Intelligence and Neuroscience*, *2011*, 156869. doi: 10.1155/2011/156869 21253357 PMC3021840

[CIT0034] Panzeri, S., Senatore, R., Montemurro, M. A., & Petersen, R. S. (2007). Correcting for the sampling bias problem in spike train information measures. *Journal of Neurophysiology*, *98*(3), 1064–1072. doi: 10.1152/jn.00559.2007 17615128

[CIT0035] Park, H., Ince, R. A., Schyns, P. G., Thut, G., & Gross, J. (2015). Frontal top-down signals increase coupling of auditory low-frequency oscillations to continuous speech in human listeners. *Current Biology*, *25*(12), 1649–1653. doi: 10.1016/j.cub.2015.04.049 26028433 PMC4503802

[CIT0036] Park, H., Kayser, C., Thut, G., & Gross, J. (2016). Lip movements entrain the observers’ low-frequency brain oscillations to facilitate speech intelligibility. *Elife*, *5*. doi: 10.7554/eLife.14521 PMC4900800 27146891

[CIT0037] Pefkou, M., Arnal, L. H., Fontolan, L., & Giraud, A. L. (2017). θ-Band and β-band neural activity reflects independent syllable tracking and comprehension of time-compressed speech. *The Journal of Neuroscience*, *37*(33), 7930–7938. doi: 10.1523/JNEUROSCI.2882-16.2017 28729443 PMC6596908

[CIT0038] Pernet, C. R., Wilcox, R., & Rousselet, G. A. (2013). Robust correlation analyses: False positive and power validation using a new open source matlab toolbox. *Frontiers in Psychology*, *3*, 606. doi: 10.3389/fpsyg.2012.00606 23335907 PMC3541537

[CIT0039] Pickering, M. J., & Garrod, S. (2007). Do people use language production to make predictions during comprehension? *Trends in Cognitive Sciences*, *11*(3), 105–110. doi: 10.1016/j.tics.2006.12.002 17254833

[CIT0040] Pickering, M. J., & Garrod, S. (2013). An integrated theory of language production and comprehension. *Behavioral and Brain Sciences*, *36*(4), 329–347. doi: 10.1017/S0140525X12001495 23789620

[CIT0041] Poeppel, D. (2003). The analysis of speech in different temporal integration windows: Cerebral lateralization as ‘asymmetric sampling in time’. *Speech Communication*, *41*, 245–255. doi: 10.1016/S0167-6393(02)00107-3

[CIT0042] Rohenkohl, G., & Nobre, A. C. (2011). Alpha oscillations related to anticipatory attention follow temporal expectations. *Journal of Neuroscience*, *31*(40), 14076–14084. doi: 10.1523/JNEUROSCI.3387-11.2011 21976492 PMC4235253

[CIT0043] Ruspantini, I., Saarinen, T., Belardinelli, P., Jalava, A., Parviainen, T., Kujala, J., & Salmelin, R. (2012). Corticomuscular coherence is tuned to the spontaneous rhythmicity of speech at 2–3 Hz. *Journal of Neuroscience*, *32*(11), 3786–3790. doi: 10.1523/Jneurosci.3191-11.2012 22423099 PMC6703459

[CIT0044] Schreiber, T. (2000). Measuring information transfer. *Physical Review Letters*, *85*(2), 461–464. doi:10.1103/PhysRevLett.85.461 doi: 10.1103/PhysRevLett.85.461 10991308

[CIT0045] Smith, Z. M., Delgutte, B., & Oxenham, A. J. (2002). Chimaeric sounds reveal dichotomies in auditory perception. *Nature*, *416*(6876), 87–90. doi: 10.1038/416087a 11882898 PMC2268248

[CIT0046] Tree, J. E. F. (1995). The effects of false starts and repetitions on the processing of subsequent words in spontaneous speech. *Journal of Memory and Language*, *34*(6), 709–738. doi: 10.1006/jmla.1995.1032

[CIT0047] Tree, J. E. F. (2001). Listeners’ uses of um and uh in speech comprehension. *Memory & Cognition*, *29*(2), 320–326. doi: 10.3758/BF03194926 11352215

[CIT0048] Tzourio-Mazoyer, N., Landeau, B., Papathanassiou, D., Crivello, F., Etard, O., Delcroix, N., … Joliot, M. (2002). Automated anatomical labeling of activations in SPM using a macroscopic anatomical parcellation of the MNI MRI single-subject brain. *Neuroimage*, *15*(1), 273–289. doi: 10.1006/nimg.2001.0978 11771995

[CIT0049] Weber, I., Florin, E., von Papen, M., & Timmermann, L. (2017). The influence of filtering and downsampling on the estimation of transfer entropy. *PLoS One*, *12*(11), e0188210. doi: 10.1371/journal.pone.0188210 29149201 PMC5693301

[CIT0050] Wostmann, M., Herrmann, B., Wilsch, A., & Obleser, J. (2015). Neural alpha dynamics in younger and older listeners reflect acoustic challenges and predictive benefits. *Journal of Neuroscience*, *35*(4), 1458–1467. doi: 10.1523/JNEUROSCI.3250-14.2015 25632123 PMC6795262

[CIT0051] Zion Golumbic, E., Cogan, G. B., Schroeder, C. E., & Poeppel, D. (2013). Visual input enhances selective speech envelope tracking in auditory cortex at a “cocktail party”. *Journal of Neuroscience*, *33*(4), 1417–1426. doi: 10.1523/JNEUROSCI.3675-12.2013 23345218 PMC3711546

