# Supplementary material for: Predictive entrainment of natural speech through two fronto-motor top-down channels
Source: Lang Cogn Neurosci. 2018 Sep 26;35(6):739–51. doi: 10.1080/23273798.2018.1506589 (PMC7446042; doi:10.1080/23273798.2018.1506589)
Supplement: PLCP_A_1506589_Supplemental Material [file PLCP_A_1506589_SM5615.docx]

**Supplemental Materials**

**
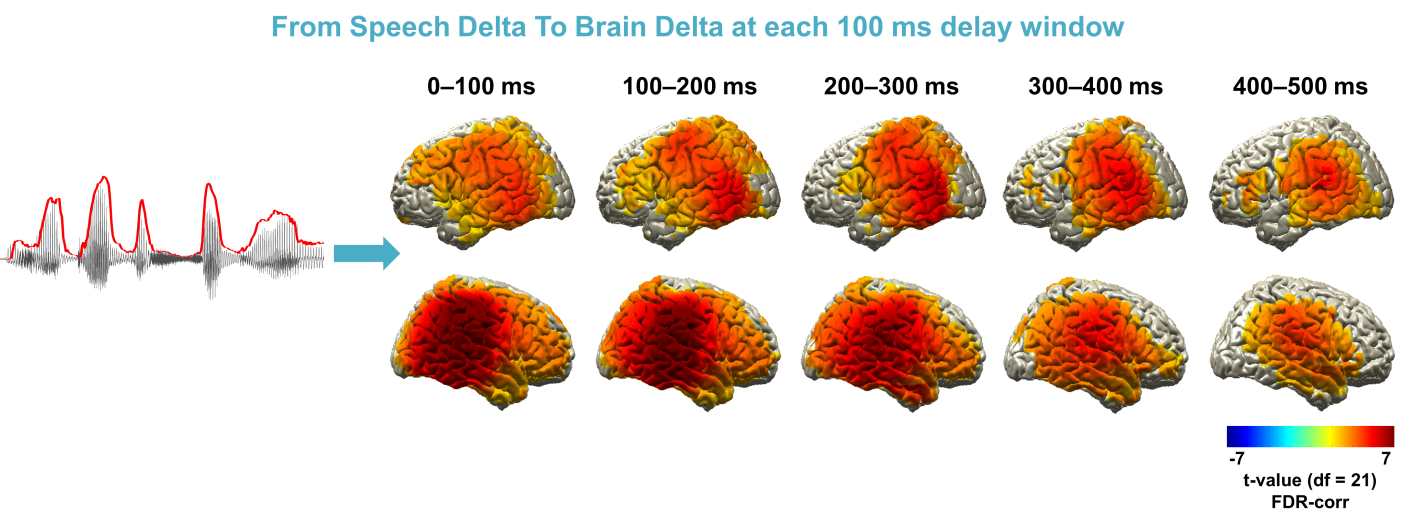
**

**Supplemental Figure 1 (related to Figure 2). Entrained brain signals following speech at whole brain at each 100 ms window.**

TE computation was performed for each condition (forward and backward played speech) at each voxel from 20 ms to 500 ms with a 20-ms step. To characterise the spatio-temporal pattern across delays at whole brain level, we averaged TE-maps in 100 ms windows and computed again the statistical contrast of forward compared to backward speech (p < 0.05, FDR-corrected). Delta phase information in the brain following the same frequency information in the speech envelope is robust across all delays and the pattern is stronger in the right hemisphere (higher t-values).


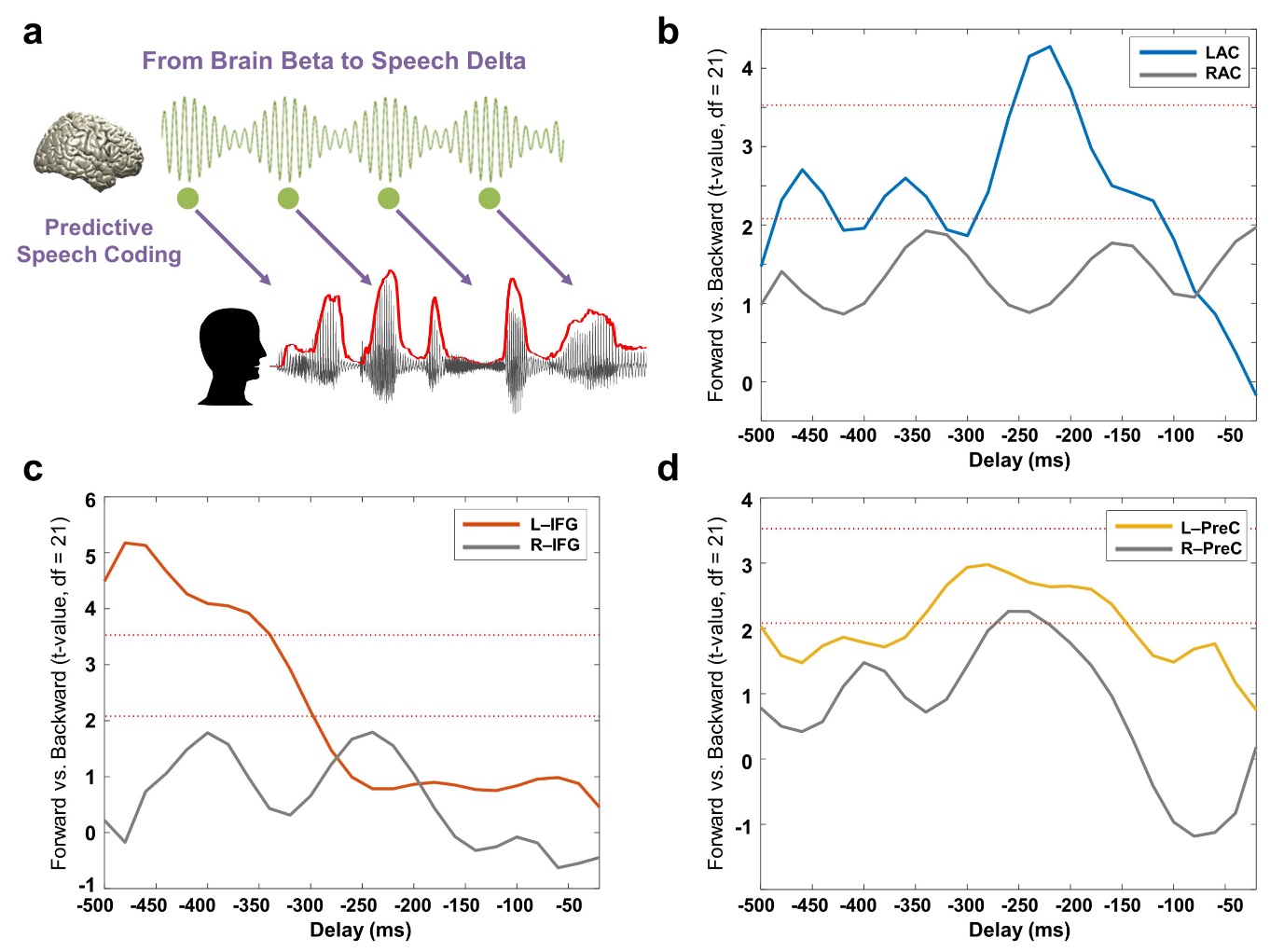


**Supplemental Figure 2 (related to Figure 4). Beta rhythms involving the prediction of upcoming speech are left-lateralised.**

Here we show the same plots as in the Figure 4b, but separately for each ROI with the homologous ROI in the right hemisphere. Green line represents beta rhythm in the brain and each circle represents a certain point in time. (a) Primary auditory cortex (Heschl gyrus), (b) Inferior frontal gyrus – opercular part, (c) Precentral gyrus. Top-down prediction by beta power in the brain to speech delta phase is left-lateralised (statistics by paired t-test; upper red line: t_21_ = 3.53, p < 0.05, corrected; bottom red line: t_21_ = 2.08, p < 0.05, uncorrected). Our results indicate three different mechanisms in terms of hemispheric asymmetry. 1) Speech-driven entrainment by delta phase is shown bilaterally (Figure 2; Supplementary Figure 1). 2) However, top-down prediction by the same delta phase shows progression from left inferior fronto-motor areas (200-300 ms prior to speech) to right auditory-temporal areas (0-200 ms prior to speech). 3) Top-down prediction by beta power in the fronto-motor areas is left-lateralised from early stage (200-500 ms prior to speech).
